# Characterization of EpCAM in thyroid cancer biology by three-dimensional spheroids in vitro model

**DOI:** 10.1186/s12935-024-03378-2

**Published:** 2024-06-04

**Authors:** Viola Ghiandai, Elisa Stellaria Grassi, Giacomo Gazzano, Laura Fugazzola, Luca Persani

**Affiliations:** 1 Department of Endocrine and Metabolic Diseases, Istituto Auxologico Italiano IRCCS, Milan, Italy; 2 Department of Medical Biotechnology and Translational Medicine (BIOMETRA), Università degli Studi di Milano, Milan, Italy; 3 Department of Pathophysiology and Transplantation, Università degli Studi di Milano, Milan, Italy; 4 Pathology Unit, Istituto Auxologico Italiano IRCCS, Milan, Italy

**Keywords:** Thyroid cancer, Tumor-initiating cells, EpCAM, Spheroid cultures, Regulated intramembrane proteolysis, Drug resistance

## Abstract

**Background:**

Thyroid cancer (TC) is the most common endocrine malignancy. Nowadays, undifferentiated thyroid cancers (UTCs) are still lethal, mostly due to the insurgence of therapy resistance and disease relapse. These events are believed to be caused by a subpopulation of cancer cells with stem-like phenotype and specific tumor-initiating abilities, known as tumor-initiating cells (TICs). A comprehensive understanding of how to isolate and target these cells is necessary. Here we provide insights into the role that the protein Epithelial Cell Adhesion Molecule (EpCAM), a known TICs marker for other solid tumors, may have in TC biology, thus considering EpCAM a potential marker of thyroid TICs in UTCs.

**Methods:**

The characterization of EpCAM was accomplished through Western Blot and Immunofluorescence on patient-derived tissue samples, adherent cell cultures, and 3D sphere cultures of poorly differentiated thyroid cancer (PDTC) and anaplastic thyroid cancer (ATC) cell lines. The frequency of tumor cells with putative tumor-initiating ability within the 3D cultures was assessed through extreme limiting dilution analysis (ELDA). EpCAM proteolytic cleavages were studied through treatments with different cleavages’ inhibitors. To evaluate the involvement of EpCAM in inducing drug resistance, Vemurafenib (PLX-4032) treatments were assessed through MTT assay.

**Results:**

Variable EpCAM expression pattern was observed in TC tissue samples, with increased cleavage in the more UTC. We demonstrated that EpCAM is subjected to an intense cleavage process in ATC-derived 3D tumor spheres and that the 3D model faithfully mimics what was observed in patient’s samples. We also proved that the integrity of the protein appears to be crucial for the generation of 3D spheres, and its expression and cleavage in a 3D system could contribute to drug resistance in thyroid TICs.

**Conclusions:**

Our data provide novel information on the role of EpCAM expression and cleavage in the biology of thyroid TICs, and our 3D model reflects the variability of EpCAM cleavage observed in tissue samples. EpCAM evaluation could play a role in clinical decisions regarding patient therapy since its expression and cleavage may have a fundamental role in the switch to a drug-resistant phenotype of UTC cells.

**Supplementary Information:**

The online version contains supplementary material available at 10.1186/s12935-024-03378-2.

## Background

Thyroid cancer (TC) is the most common endocrine malignancy, with a frequent favorable outcome. However, undifferentiated thyroid cancers (UTCs) are still lethal, mostly because of the appearance of therapy resistance and disease relapse. Like in many other neoplasms [1–5], this is believed to be caused by a subpopulation of cancer cells with stem-like phenotype and tumor-initiating abilities, known as tumor-initiating cells (TICs), that usually reside in specific microenvironmental niches. This term represents a functional definition of the capacity of these cells to induce tumor formation in xenotransplantation studies. To date, many in vivo [6] and in vitro assays [7–9] have been applied for the identification of TICs, as well as variations of enzymatic activities [10], expression of stemness [11, 12] or surface markers [13–15]. Epithelial Cell Adhesion Molecule (EpCAM; CD326) belongs to the latter category; it is a highly conserved type 1 transmembrane glycoprotein that was discovered during screenings against antigens derived from colorectal cancer cells [16]. EpCAM has already been defined as a marker for TICs in several human epithelial tumors, including breast [17], ovarian [18], pancreatic [19], urothelial [20] and gallbladder cancer [21], however it is poorly studied in TC. In specific tumor microenvironmental niches or as a result of the loss of tumor cells differentiation, EpCAM may be subjected to a proteolytic process called regulated intramembrane proteolysis (RIP) that involves ADAM17 and γ-secretase containing presenilin-2 (PS-2) [22] at the plasma membrane, while β-secretase can induce an alternative intracellular cleavage [23]. These cleavages result in the shedding of the extracellular domain (EpEx), which may act as a ligand for epidermal growth factor receptor (EGFR) [24], and the consecutive release of the intracellular domain (EpICD) into the cytoplasm from where it may translocate into the nucleus and acts as regulator of cell division [25], epithelial-to-mesenchymal transition (EMT) [26] and pluripotency [27]. Most of the studies of EpCAM in TC biology focus on evaluating its expression in patient-derived tissues by immunohistochemistry [28–31]. In particular, Ralhan et al. demonstrated that loss of membranous expression of EpCAM and increased cytoplasmic and nuclear accumulation of the EpICD domain correlate with tumor aggressiveness and poor prognosis [28, 29]. These data were also confirmed by a couple of studies performed on TC immortalized cell lines that identified EpCAM as a marker of cells with stem-like phenotype, but without investigating the mechanisms at the basis of EpCAM RIP induction and its role in the maintenance of the stem-like phenotype [15, 30]. In the present work, we provide insights into the role that EpCAM may have in TC biology and therapy response through the characterization of its expression and cleavage pattern in patient-derived tissue samples, TC immortalized cell lines and three-dimensional (3D) spheroids.

## Methods

### Patients and sample collection

Patient-derived tissue samples were used after institutional review board approval (Ethical Committee of the IRCCS Istituto Auxologico Italiano: 05C212 THY-CANC) and informed consent for the use of thyroid tumor tissues was obtained from all participants. Frozen thyroid tissues from 15 patients undergoing total thyroidectomy were used for Western Blot and Immunofluorescence analysis. We evaluated 4 papillary (PTC), 3 follicular (FTC), 4 poorly differentiated (PDTC) thyroid cancers, and 4 normal thyroid tissues. Tumors were classified and staged according to the thyroid malignancy World Health Organization classification and the 8th edition of TNM staging [32].

### Cell cultures

B-CPAP (PDTC), SW1736 (anaplastic thyroid cancer, ATC), HTCC3 (ATC) and human embryonic kidney (HEK) 293T cells were grown in DMEM (Gibco); SW579 (PDTC of squamous TC origin) and FTC133 (metastatic aggressive FTC) cells were grown in DMEM/F12 (Gibco); FRO (ATC) cells were grown in RPMI supplemented with L-Glutamine (Euroclone) in a humidified incubator at 37 °C under 5% CO2. All media were supplemented with 10% fetal bovine serum (Sigma Aldrich) and penicillin-streptomycin mixture (Sigma Aldrich). All experiments were performed with cell lines in between the 8th and 18th passage.

- PDTC and ATC cell lines were kindly provided by Dr. I. Bongarzone (Istituto Nazionale dei Tumori, Milan, Italy). HEK293T cell line was kindly gifted by Professor V. Silani (Istituto Auxologico Italiano, Milan, Italy).
- All cell lines were authenticated by short tandem repeat (STR) profiling.
- All cell lines were routinely screened for mycoplasma contamination with Venor®GeM Classic Mycoplasma Detection Kit (Minerva Biolabs®).

### Three-dimensional (3D) spheroids generation and clonality assay

Three-dimensional spheroids were generated from immortalized TC cell lines. For the hanging-drop technique, cells were seeded at clonal density through serial dilutions (20, 10, 5, 1 cell per 33 µl drop). The drops were directly spotted on the inner surface of a 48-well plate lid. Cells were kept in a humidified incubator at 37 °C, 5% CO2 for 7–10 days until 3D spherical structures appeared. 3D spheres were obtained after seeding cells in their appropriate culture medium or in Sphere Medium (DMEM/F12 (1:1) Nutrient Mixture (Ham)), supplemented with human EGF (PeproTech EC) 20 ng/ml, human b-FGF (PeproTech EC) 20 ng/ml, and B-27™ Supplement 1:50 (Thermo Fisher Scientific), previously used to isolate cancer stem-like cells (CSCs) in primary cultures [33]. 3D spheres generation using poly(2-hydroxyethyl methacrylate) non-adhesive substrate was performed as previously described, with cells seeded at 25,000 cells/ml [34]. Light microscopy for 3D spheres dimensions and morphology is described in Additional file 1. For clonality evaluation, cells were seeded at different densities through serial dilutions and 3D spheres formation was evaluated with the hanging-drop technique. After 7–10 days the number of wells containing spheres was counted and extreme limiting dilution analysis (ELDA) was performed with the online tool [35]. Limiting dilution analysis (LDA) assay is an experimental technique that quantifies the proportion of biologically active particles in a larger population and is a commonly applied in stem cell research. LDA presumes the Poisson single-hit model, which assumes that the number of biological active particles in each culture varies according to a Poisson distribution, and a single biologically active cell is sufficient for a positive response from a culture. Extreme limiting dilution analysis (ELDA) assay is a further evolution of LDA for extreme data situations, non-Poisson distribution and multiple populations.

### Proteins extraction and Western blotting

Tissue lysates were prepared in Laemmli buffer as previously reported [36], while adherent cells were lysed in ice-cold RIPA buffer with protease and phosphatase inhibitors (Roche) as previously described [37]. Three-dimensional spheres were collected and lysed directly by resuspension in ice-cold RIPA, sonicated, and protein amount was dosed with the Pierce BCA protein Assay Kit (Thermo Fisher Scientific). Protein extracts were separated on NuPage 10% Bis-Tris Gels (Thermo Fisher Scientific) and transferred with iBlot Dry Blotting System (Thermo Fisher Scientific). The primary and secondary antibodies used are described in Additional file 3, Table S1. The antibodies used for EpCAM recognition are Anti-EpCAM antibody (E144) directed against an epitope localized in the ICD (EpICD), able to recognize both full length and intracellular cleaved fragments, and EpCAM/TROP-1 Antibody (AF960) that recognizes the full length form but not the ICD (EpEX). A chemiluminescence-based immunodetection was performed with ECL Star Detection (Euroclone) or with Westar Supernova ECL Substrate (Cyanagen) and images were acquired with c400 camera (Azure Biosystems). Band intensity was quantified with FIJI software.

### Immunofluorescence

Immunofluorescence experiments on tissue sections and adherent cells were performed as previously described [38, 39]. Coating with cold Corning™ Membrane Matrix Matrigel™ (Thermo Fisher Scientific) was used for Immunofluorescence on 3D spheres. 10 µl of Matrigel were mixed with 1 ml of ice-cold culture medium without FBS and penicillin/streptomycin. The 3D spheres obtained in poly-hema plates were collected, gently centrifuged, seeded on Matrigel-coated coverslips, and let attach for 2/3 hours. The Immunofluorescence protocol for tissues, adherent cells and 3D spheres is described in Additional file 3, Table S2A. The primary and secondary antibodies used are described in Additional file 3, Table S2B, C. Fluorescence and confocal microscopy acquisitions are described in Additional file 1. To determine the cell epithelial or mesenchymal phenotype, nucleus circularity (C_N_) and the Aspect Ratio (A_R_) of the cell were determined with FIJI software. A value close to 1 is attributed to an epithelial, cobblestone-like morphology, whereas values closer to 0 describe a spindle-like appearance [40].

### EpCAM cleavages’ inhibitors and pseudo-hypoxic treatments effects on sphere-forming abilities

Treatments were performed 48 hours after seeding FRO as adherent cells (200000 cells/well) in a 6-well plate. Cells were treated with selected concentrations of EpCAM cleavages’ inhibitors: TAPI-2 (ADAM17 inhibitor, Merck Millipore) [41–43], BACE (β-secretase inhibitor, Merck Millipore) [44] and DAPT (γ-secretase inhibitor, Merck Millipore) [41, 45, 46]. Increasing concentrations of 2,2’-Bipyridyl (DIP) (Merck Millipore) were used to induce pseudo-hypoxia [41, 47, 48]. Cells were collected and seeded at clonal density as hanging-drops (10 cells per drop) 24 h after treatments. The ability to generate 3D spheres was assessed after 7–10 days of culture.

### Vemurafenib (PLX-4032) treatment

Cells were seeded (1500 cells/well for FRO, 3000 cells/well for HTCC3 and 2000 cells/well for FTC133) in 96-well plates and treated with increasing concentrations (0-100 µM) of Vemurafenib (PLX-4032, Selleckchem). Cell proliferation was assessed after 72 h with Thiazolyl Blue (MTT, MedChemExpress). Formazan crystals were solubilized in 200 µl of EtOH-DMSO 1:1 solution. For treatment on 3D spheres, 10 µl of MTT solution were added to each well and incubated at 37˚C for 1 h. The spheres were collected and directly resuspended in 200 µl of EtOH: DMSO solution. Absorbance was red at 540 nm using the ELx800 Absorbance Microplate Reader.

### Statistical analysis

All Western Blot quantifications were made with Fiji Software. Confocal microscopy quantifications were made with Nikon NIS-Elements AR Software, followed by Fiji analysis. All statistical analyses were performed with GraphPad Prism Software version 9 on a minimum of three independent experiments. Values are expressed as mean ± standard error of the mean (SEM). One-way and 2way ANOVA and non-parametric tests followed by the appropriate post-hoc test were used, as indicated in the figure legends.

## Results

### EpCAM is expressed and variably cleaved in healthy and neoplastic thyroid tissues

**Fig. 1:**
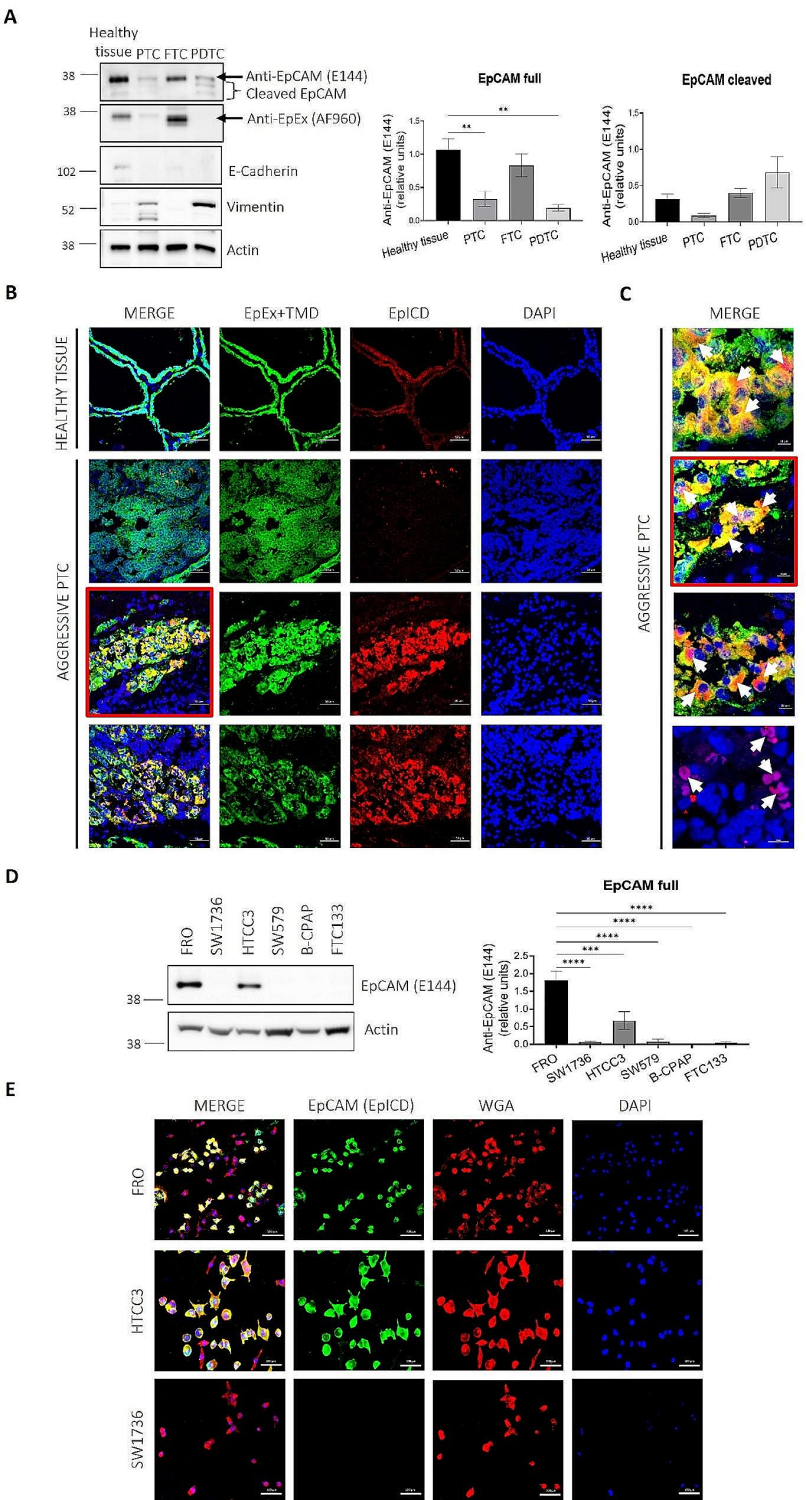
EpCAM is subjected to differential expression and cleavage in tissue samples and TC cell lines. (**A**) Western Blot representative images and corresponding quantifications of 4 healthy thyroid, 4 PTC, 3 FTC and 4 PDTC tissue samples. EpCAM expression was evaluated with Anti-EpCAM antibody (E144) directed against an epitope localized in the ICD (EpICD), that recognizes both full length (arrow) and intracellular cleaved fragments (bracket), and EpCAM/TROP-1 Antibody (AF960) that detects the full length form but not the ICD (EpEX). E-Cadherin was used as epithelial marker, Vimentin as mesenchymal marker, Actin as loading control. Statistical analysis: One way ANOVA followed by Dunnett’s multiple comparisons test. **p < 0.01. PTC = papillary thyroid cancer; FTC = follicular thyroid cancer; PDTC = poorly differentiated thyroid cancer. (**B**) Confocal microscopy representative images of tissue cryosections of healthy thyroid and aggressive PTC, characterized by undifferentiated regions, showing expression of EpEx + TMD (EpCAM/TROP-1 Antibody AF960) in green, EpICD (Anti-EpCAM antibody E144) in red and DAPI staining for nuclei in blue. Scalebars 50 μm. EpEx = extracellular domain of EpCAM; TMD = transmembrane domain of EpCAM; EpICD = intracellular domain of EpCAM. (**C**) Higher magnification (60X) images representative of the different EpCAM signal observed in (**B**). Only the second image from the top is an higher magnification of one of the aggressive PTC images (red boxes). Nuclear and cytoplasmic staining for EpICD is indicated by white arrows. Scalebars 10 μm. (**D**) Western Blot of protein extracts obtained from FRO, SW1736, HTCC3, SW579, B-CPAP and FTC133 cultured as adherent cells. Actin was used as loading control. Statistical analysis: One-Way ANOVA followed by Dunnet’s multiple comparisons test. ***p < 0.001, ****p < 0.0001. (**E**) Immunofluorescence of FRO, HTCC3 and SW1736 adherent cells showing EpCAM + and EpCAM- subpopulations in FRO (61,9% ± 5,9 EpCAM+; 38,1% ± 5,9 EpCAM-) and HTCC3 (77,8% ± 10,44 EpCAM+; 22,2% ± 10,44 EpCAM-). Scalebars 100 μm. EpCAM + cells in green (EpICD), plasma membrane marker Wheat Germ Agglutinin (WGA) in red and DAPI for nuclei in blue

EpCAM expression and cleavage in thyroid cancer were evaluated on different tumor histotypes from a cohort of TC patients (Table 1). For determining the cleavage rate, we used two different antibodies, Anti-EpCAM antibody (E144) directed against an epitope localized in the ICD (EpICD), able to recognize both full length and intracellular cleaved fragments, and EpCAM/TROP-1 Antibody (AF960) that recognizes the full length form but not the ICD (EpEX). Our results show a similar expression pattern of EpCAM in healthy tissues, with a strong signal of the full-length form and extremely faint bands with lower molecular weight products, deriving from EpCAM cleavage (Fig. 1A). Despite wide inter-patient variability, EpCAM full length signal in FTCs was similar to that in healthy tissues, while a significant decrease was observed in PTCs and PDTCs (Fig. 1A). In PDTC samples, bands with lower molecular weight referable to EpCAM cleavage products were visible. This was associated with a strong increase in Vimentin, a well-known mesenchymal marker, and loss of E-Cadherin, a typical epithelial marker (Fig. 1A). Differently, in PTC we detected a general decrease of full length EpCAM, EpCAM cleavage and E-cadherin levels, together with increased Vimentin cleavage, probably indicating the presence of other biological processes such as early apoptosis and inflammation [49, 50]. Consistently, confocal microscopy experiments on tissue cryosections showed a constant EpCAM signal in healthy thyroid and high variability in EpCAM expression and subcellular localization in undifferentiated areas of the same tumor sample (aggressive PTC) (Fig. 1B). We noticed areas of EpEx and transmembrane domain (EpEx + TMD) uniform expression at the plasma membrane with almost total absence of EpICD signal, as well as regions with a variable expression of both EpEx + TMD and EpICD (Fig. 1B). In particular, high cytoplasmic and/or nuclear signal of EpICD with a variable EpEX + TMD signal or an almost total loss of EpEX + TMD signal with only nuclear localization of EpICD was observed (Fig. 1C).

**Table 1:** Cohort of TC patients representative of the different histotypes

Patient	Gender	Genetic	Histotype	Patient age at diagnosis (year old)	TNM^a^ classification	AJCC^b^ 8th Stage at diagnosis
#1	M	BRAF V600E	PTC^c^	35	T = pT4a, N = N1a, M = no	I
#2	F	BRAF V600E	PTC	39	T = pT1b, N = N1a, M = no	I
#3	F	BRAF V600E	aggressive PTC	69	T = T2, N = N0, M = n.d.^d^	I
#4	M	BRAF V600E + RET/PTC	PTC	64	T = pT1b, N = N1b, M = no	II
#5	M	RAS	FTC^e^	47	T = T1b, N = N1a, M = yes	I
#6	F	NRAS Q61R + TERT	FTC	81	T = T4a, N = NX, M = yes	IVB
#7	M	n.d.	aggressive FTC	80	T = T3, N = N0, M = M1	IVB
#8	F	TERT + P53	PDTC^f^	n.d.	n.d.	n.d.
#9	F	BRAF V600E + TERT	PDTC	72	T = pT4, N = N1b, M = no	IVA
#10	M	PTEN mut	PDTC	34	T = pT2, N = NX, M = yes	I
#11	F	P53	PDTC	n.d.	n.d.	n.d.

TNM^a^ = Tumor, Nodes, Metastasis; AJCC^b^ = American Joint Committee on Cancer; PTC^c^ = papillary thyroid cancer; n.d.^d^ = not determined; FTC^e^ = follicular thyroid cancer; PDTC^f^ = poorly differentiated thyroid cancer

### TC cell lines present different EpCAM expression levels

The screening of six TC cell lines (FRO, SW1736, HTCC3, SW579, B-CPAP and FTC133) by Western Blot revealed that FRO, and in small amount HTCC3, retain full length EpCAM expression in basal conditions (Fig. 1D), while the other cell lines do not express the protein. Immunofluorescence experiments performed on adherent cells confirmed this result. Wheat germ agglutinin (WGA) was used to label all cell membranes, while to identify EpCAM-expressing cells we used the Anti-EpCAM antibody (E144). The results revealed that EpCAM expression was not uniformly distributed among cells but two subpopulations were observed, one EpCAM positive (EpCAM+) and one EpCAM negative (EpCAM-) both in FRO (61,9% ± 5,9 EpCAM+; 38,1% ± 5,9 EpCAM-) and in HTCC3 (77,8% ± 10,44 EpCAM+; 22,2% ± 10,44 EpCAM-) (Fig. 1E). Immunofluorescence experiments on SW1736 cells confirmed the absence of EpCAM expression as observed at Western Blot.

### TC cell lines present different 3D sphere-forming abilities

**Fig. 2:**
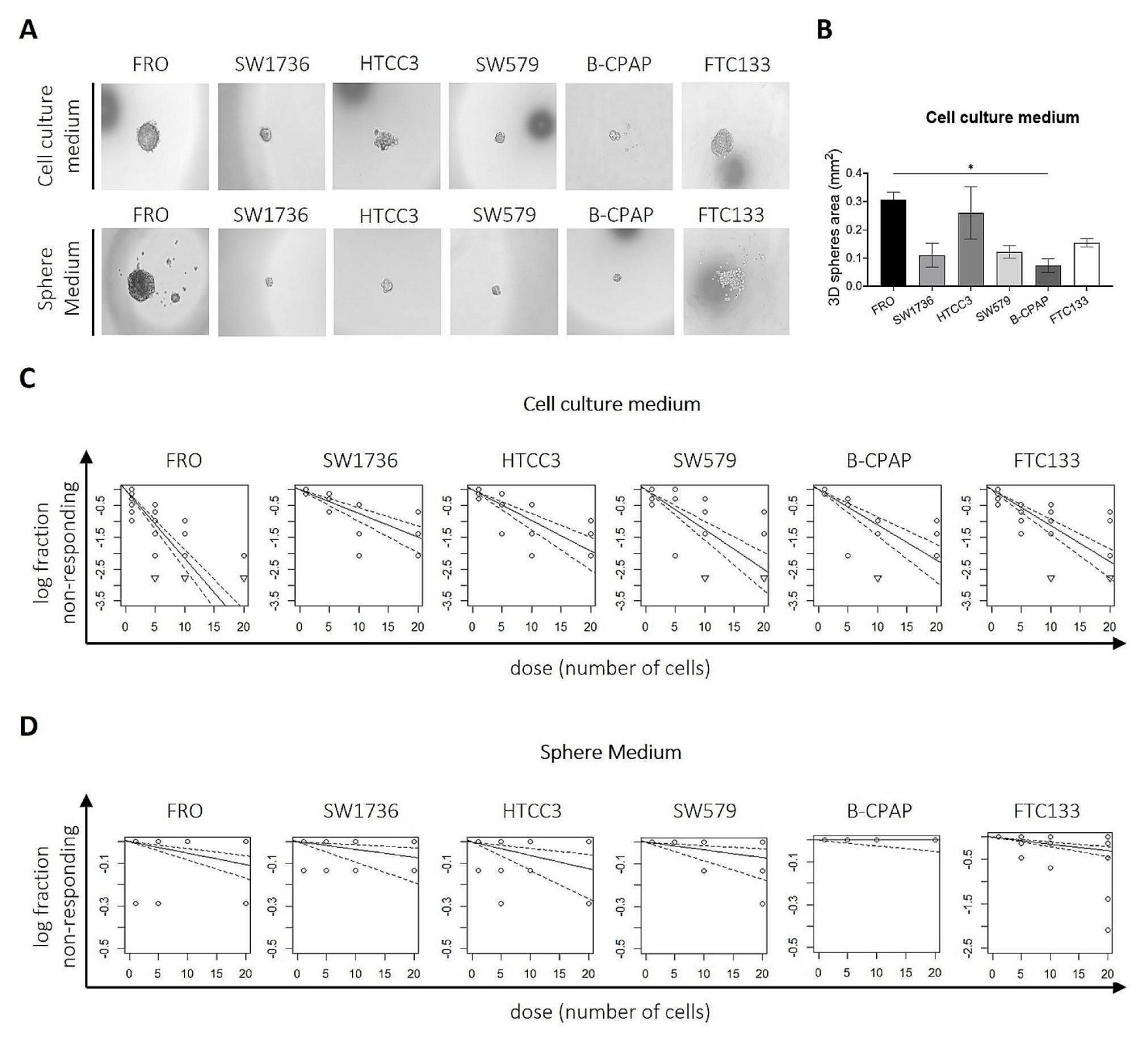
TC cell lines have different 3D sphere-forming abilities. (**A**) Representative morphological panel of spheres obtained from different TC cell lines when 10 cells per drop were seeded in their culture medium (upper panel) or in Sphere Medium (lower panel). (**B**) Sphere’s dimensions quantification: for each cell line, an average of the measurements of the area of at least 7 spheres for each independent replicate is reported. The area of each sphere was calculated on Fiji applying the ellipses formula. The number of replicates for each cell line is reported as follows: FRO *n* = 13, SW1736 *n* = 4, HTCC3 *n* = 4, SW579 *n* = 5, B-CPAP *n* = 4, FTC133 *n* = 7. Statistical analysis: ANOVA Kruskal-Wallis followed by Dunn’s multiple comparisons test. *p < 0.05. (**C**, **D**) Extreme Limiting Dilution Analysis (ELDA) of 3D spheres obtained after seeding cells in their culture medium (**C**) and in Sphere Medium (**D**). The number of replicates for each cell line is the same as for (**B**). The x-axis shows the values of the dose (i.e., the number of cells seeded), the y-axis shows the logarithmic value of the non-responding fraction of cells. The closer the trendlines are to the y-axis, the greater is the positivity of ELDA analysis

Since one of the most valid approaches to study thyroid TICs in vitro is to evaluate their self-renew as sphere-forming ability when seeded at clonal density, we tested the ability of our TC cell lines to generate 3D spheres. We observed that all cell lines were able to originate clonal 3D spheres, with a strong variability in number, morphology and dimensions (Fig. 2A, B). Very few 3D structures were obtained in Sphere Medium, characterized by more irregular shapes and smaller dimensions than those obtained in the original culture medium. The only cell lines that displayed more rounded-shape morphology and larger dimensions in both media were FRO and FTC133 (Fig. 2A). ELDA analysis performed in all the cell lines revealed a general impairment in cells ability to generate 3D spheres when seeded in Sphere Medium, probably because immortalized cell lines have very low growth abilities in absence of serum. However, cells with self-renew and tumor-initiating abilities could be selected through the seeding at clonal density in their culture medium. By the analysis we observed that FRO cells may have the greatest frequency of tumor cells with potential tumor-initiating ability (1 out of 5.16) respect to the other cell lines when cells are cultured in their culture medium (Fig. 2C; Tables 2 and 3). When cells are seeded in Sphere Medium, we detected high variability between the different cell lines, with the lowest ratio represented by FTC133, characterized by an estimate value of 1 out of 67 (Fig. 2D; Tables 2 and 3).

**Table 2:** Extreme Limiting Dilution Analysis (ELDA) estimated confidence intervals for 1/tumor-initiating ability

	Culture Medium			Sphere Medium		
Cell line	Lower	Estimate	Upper	Lower	Estimate	Upper
B-CPAP	12.2	9.53	7.44	Inf	Inf	385
FRO	5.85	5.16	4.56	299	188	118
FTC133	11.3	9.36	7.76	97.5	67	46.2
HTCC3	14	10.8	8.42	338	160	76.2
SW1736	18.2	13.9	10.6	759	284	106
SW579	10.6	8.51	6.84	669	279	117

ELDA webtool was applied to perform the analysis. For each cell line, the confidence intervals for frequency of tumor cells with potential tumor-initiating ability were computed by ELDA online tool by entering the main parameters required for the analysis: the number of cells cultured (dose), the number of wells tested for each condition (tested) and the number of wells where the generation of 3D spheres occurred (response)

**Table 3:** ELDA pairwise tests for differences in tumor-initiating abilities

Cell line (group 1)	Cell line (group 2)	Culture medium Pr(> Chisq)	Sphere Medium Pr(> Chisq)
B-CPAP	FRO	0.0733	0.146
B-CPAP	FTC133	0.961	0.016
B-CPAP	HTCC3	0.716	0.115
B-CPAP	SW1736	0.304	0.226
B-CPAP	SW579	0.742	0.234
FRO	FTC133	0.0822	0.254
FRO	HTCC3	0.0331	0.885
FRO	SW1736	0.00581	0.747
FRO	SW579	0.143	0.756
FTC133	HTCC3	0.68	0.317
FTC133	SW1736	0.284	0.148
FTC133	SW579	0.78	0.153
HTCC3	SW1736	0.509	0.641
HTCC3	SW579	0.491	0.651
SW1736	SW579	0.179	0.991

Pairwise comparison tests for differences in tumor-initiating abilities between the different cell lines were computed by ELDA webtool. Pairwise comparisons between FRO and HTCC3 and FRO and SW1736 were significant (*p* = 0.0331, *p* = 0.00581 respectively) only in spheres obtained in their culture medium but not in Sphere Medium. Pairwise comparison between B-CPAP and FTC133 was significant (*p* = 0.016) only in spheres obtained in Sphere Medium

### FRO-derived 3D spheres reflect EpCAM cleavage pattern observed in patient-derived tissues

**Fig. 3:**
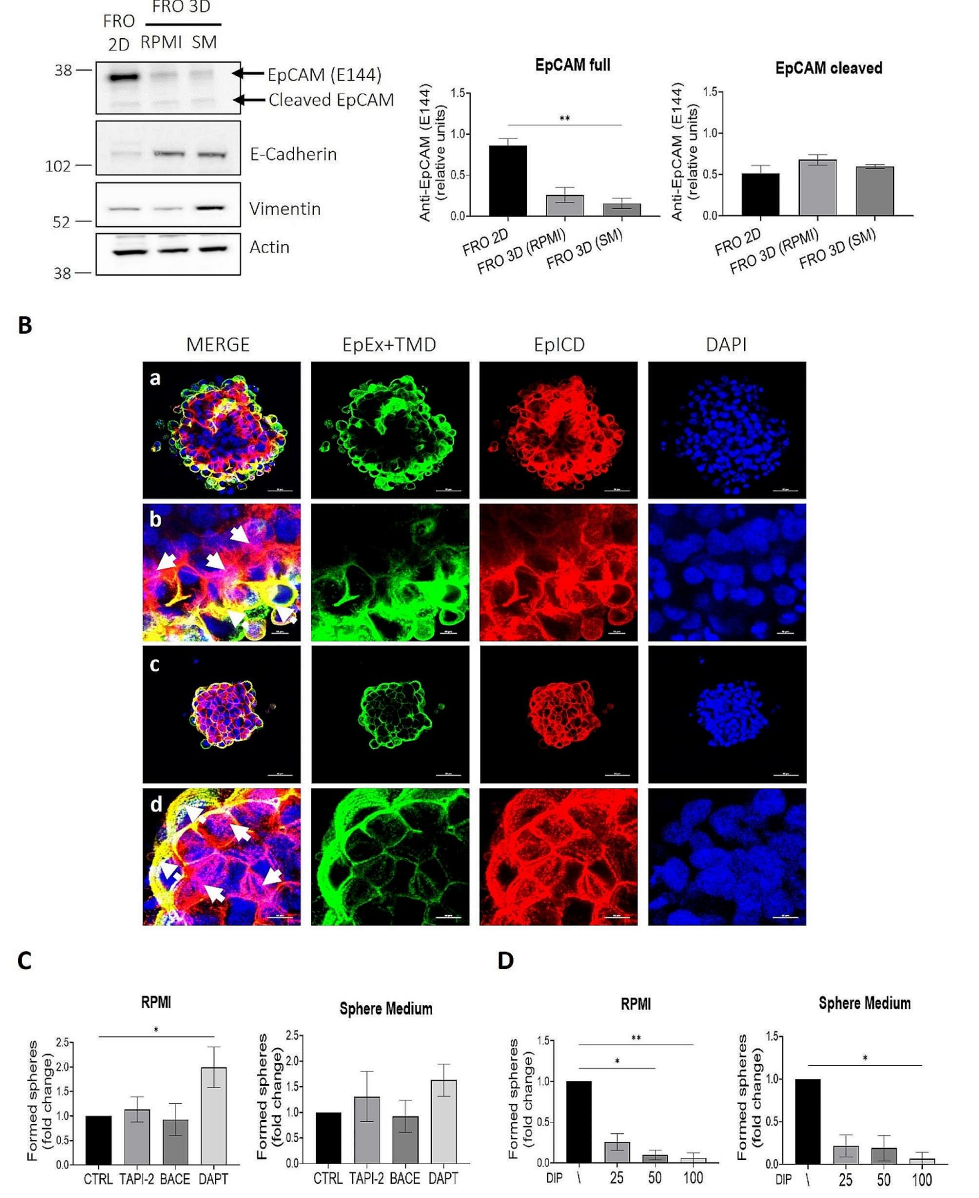
FRO-derived 3D spheres reproduce in vitro EpCAM variability observed in patient’s samples. (**A**) Western Blot of protein extracts obtained from FRO adherent cells and from FRO-derived 3D spheres. Actin was used as loading control. Statistical analysis: ANOVA Kruskal-Wallis test followed by Dunn’s multiple comparisons test. **p < 0.01. R = RPMI, SM = Sphere Medium. (**B**) Confocal microscopy representative images of 3D spheres obtained in Sphere Medium (**a**, **c**) and respective magnification of a selected area of the sphere (**b**, **d**). A strong cytoplasmic and even nuclear staining of EpICD was appreciated inside the spheres compared to EpEx + TMD that was mainly located at the plasma membrane and in the outer layer of the spheres. White arrows indicate cytoplasmic/nuclear signal of EpICD; dotted white arrows indicate membranous EpEx + TMD signal. Expression of EpEx + TMD in green, EpICD in red and DAPI staining for nuclei in blue. Scale bars (**a**, **c**) 50 μm; (**b**, **d**) 10 μm. (**C**) Effect of different EpCAM cleavage’s inhibitors on FRO sphere-forming abilities. FRO were first seeded as adherent cells and treated with TAPI-2 (20 µM), BACE (30 nM) and DAPT (10 µM) after 48 h. Cells were then collected and seeded at clonal density as hanging-drops (10 cells per drop) in RPMI and Sphere Medium, 24 h post treatments. The ability of cells to generate 3D spheres was assessed after 7–10 days of culture. (**D**) Effect of pseudo-hypoxia on FRO sphere forming abilities. FRO were first seeded as adherent cells and treated with DIP (25 µM, 50 µM, 100 µM) after 48 h. Cells were then collected and seeded at clonal density as hanging-drops (10 cells per drop) in RPMI and Sphere Medium, 24 h post treatments. The ability of cells to generate 3D spheres was assessed after 7–10 days of culture. Statistical analysis: ANOVA Kruskal-Wallis test followed by Dunn’s multiple comparisons test. *p < 0.05, **p < 0.01

Since FRO have an EpCAM positive subpopulation and high TICs abilities, we selected this cell line to study EpCAM cleavage in vitro. Western blot experiments revealed that when FRO cells are cultured as 3D spheres, a decrease in the expression of full length EpCAM, together with a slight or strong increased expression of E-Cadherin and/or Vimentin can be respectively appreciated (Fig. 3A). Confocal microscopy experiments revealed that most cell in the 3D spheres are EpCAM positive, showing a trend in EpCAM cleavage and a shift in its subcellular localization that resemble what is observed in patient-derived tumor tissues (Fig. 3B). A progressive reduction of EpEx + TMD signal was detected proceeding from the outer surface to the inner core of the spheres. This was paired to almost opposite variations in EpICD signal, displaying increased cytoplasmic and even nuclear localization in the intermediate/inner part of the spheres.

### EpCAM cleavage influences FRO sphere-forming abilities

In Additional file 2, Figure S1 we reported the complex pattern of EpCAM cleavage, by transiently transfect FRO adherent cells with a C-terminal EGFP-tagged EpCAM plasmid and by treating transfected cells with EpCAM cleavages’ inhibitors (TAPI-2, BACE and DAPT) for a better characterization of each cleavage product. Moreover, since we were able to manipulate the expression of EpCAM in response to different stimuli as shown in Additional file 2, Figure S2, where we observed that the hypoxia-mimetic compound DIP significantly decreases EpCAM full-length expression, we aimed to understand how this may influence the ability of cells with potential tumor-initiating abilities to generate the 3D spheres. Adherent FRO were treated with selected concentrations of EpCAM cleavage’s inhibitors (TAPI-2, BACE and DAPT) and with increasing concentrations of DIP. The ability of cells to generate 3D spheres was assessed after 7–10 days of culture. The sphere-forming ability of FRO in RPMI was slightly increased after ADAM17 inhibition, decreased after β-secretase inhibition and significantly increased after γ-secretase inhibition. A similar but less significant trend was observed in Sphere Medium (Fig. 3C). On the other hand, the sphere-forming ability of cells was significantly compromised after increasing concentrations of DIP; the same trend was observed both in RPMI and in Sphere Medium (Fig. 3D).

### Vemurafenib (PLX-4032) treatment

**Fig. 4:**
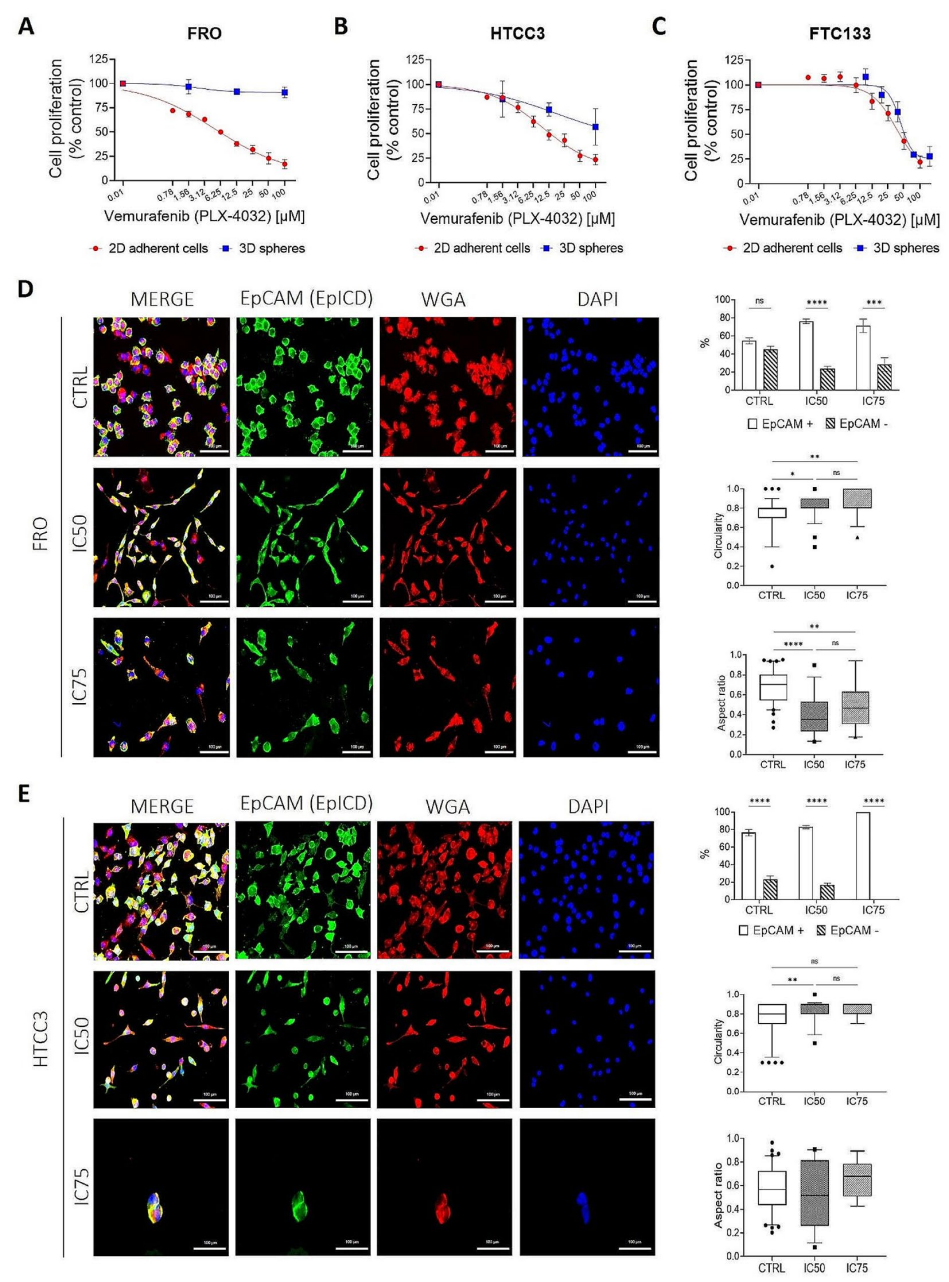
EpCAM + and EpCAM- cells show differential response to PLX-4032 treatment. (**A**-**C**) Cells cultivated as 2D adherent or 3D spheres were treated with increasing concentrations of PLX-4032 (0-100 µM) and proliferation was assessed with MTT assay (% control) 72 h after treatment. Red dots represent each data point of the dose-response curve for adherent cells; blue squares represent each data point of the dose-response curve for 3D spheres. (**D**, **E**) Immunofluorescence experiments on FRO and HTCC3 adherent cells showing different resistance to PLX-4032 of EpCAM + and EpCAM- subpopulations and quantification as relative percentage of EpCAM + and EpCAM- cells respect to the total number of cells (upper graphs). Cells were seeded and treated with PLX-4032 IC50 (6 µM for FRO and 13 µM for HTCC3) and IC75 (41 µM for FRO and 79 µM for HTCC3) 24 h later, and Immunofluorescence was performed 72 h after treatment. EpCAM + cells in green (EpICD), WGA in red and DAPI staining for nuclei in blue. Scale bars 100 μm. At least 10 different fields for each condition have been analyzed. CTRL = untreated cells. Statistical analysis: 2way-ANOVA followed by Šídák’s multiple comparisons test. ns, not significant; ****p < 0.0001; ***p = 0.0001. For the quantification of nucleus circularity (C_N_) (intermediate graphs) and the Aspect Ratio (A_R_) (lower graphs), image analysis via Fiji software is presented as box plots with 5–95 percentiles. The total number of cells analyzed are 132 for FRO and 136 for HTCC3. Statistical analysis: Kruskal-Wallis test followed by Dunn’s multiple comparisons test. ****p < 0.0001, **p < 0.005, *p < 0.05

Finally, we evaluated if EpCAM expression may play a role in drug resistance. For this purpose, we evaluated the response to Vemurafenib (PLX-4032) in EpCAM-expressing cell lines (FRO and HTCC3) and in one EpCAM negative cell line (FTC133) that shows similarly high TICs. PLX-4032 is a single target tyrosine kinase inhibitor, commonly used in clinical practice, directed mainly against BRAF-mutated cells (particularly BRAFV600E) but whose inhibitory effect has been detected in vitro also in BRAF wild-type (wt) cells. We observed that FRO, HTCC3 and FTC133 were responsive to PLX-4032 with a decrease in cell proliferation when treated as 2D adherent cells (Fig. 4A-C and Additional file 3, Table S3). Interestingly, we noticed evident differences between the 2D and 3D models only for the EpCAM-expressing cell lines (Fig. 4A, B). Since we demonstrated that both FRO and HTCC3 present EpCAM + and EpCAM- subpopulations, we hypothesized that the trend obtained from the dose-response curves may be due to a different sensitivity of the two subpopulations to PLX-4032 treatment. To validate this hypothesis, we performed Immunofluorescence on both FRO and HTCC3 adherent cells 72 h after the treatment with selected concentrations of PLX-4032. The results suggested that EpCAM + cells appear to be more resistant than EpCAM- after PLX-4032 treatment both in FRO and HTCC3 (Fig. 4D, E) and this is a common feature of tumor cells that display tumor-initiating properties. Interestingly, we also noticed that after the treatment with PLX-4032 (IC50 and IC75) cells seem to display a more elongated, “mesenchymal-like” morphology, respect to the untreated control, in both cell lines, as shown by the differences in the nucleus circularity (C_N_) and the Aspect Ratio (A_R_) of the cell. In FRO cells, we observed a significant increase of the nucleus circularity respect to the untreated control (CTRL = 0.738 ± 0.03; IC50 = 0.808 ± 0.027; IC75 = 0.847 ± 0.042), accompanied by a significant decrease of the Aspect Ratio of cell morphology in IC50 and IC75 treated cells respect to the untreated control (CTRL = 0.683 ± 0.033; IC50 = 0.398 ± 0.066; IC75 = 0.495 ± 0.099), representative of a more elongated morphology. In HTCC3, we obtained a significant increase in nucleus circularity only in IC50 treated cells respect to the untreated control (CTRL = 0.776 ± 0.032; IC50 = 0.856 ± 0.031; IC75 = 0.846 ± 0.039), but no significant differences were detected in the Aspect Ratio of cell morphology between the different conditions (CTRL = 0.576 ± 0.037; IC50 = 0.543 ± 0.092; IC75 = 0.659 ± 0.085) (Fig. 4D, E).

## Discussion

The whole of the data support the view of EpCAM expression and cleavage status as useful biomarker of thyroid tumor de-differentiation and suggest a potential role in the modulation of the response to TKIs. Our data on patient-derived tissues demonstrated that the variations of EpCAM cleavage could be associated with a different degree of differentiation of neoplastic cells within the tumor, probably induced by the same local microenvironmental alterations that support the presence of TICs and stem-like cells in specialized niches [51–54] (Fig. 1). These data are also consistent with previous literature regarding the loss of membranous expression of EpCAM and increased cytoplasmic and nuclear accumulation of the EpICD fragment detected in ATCs [28, 29]. Interestingly, we observed that EpCAM is predominantly expressed in cell lines with high frequency of potential TICs within the 3D sphere cultures. Applying the 3D model, the same variations in the degree of EpCAM cleavage obtained in tumor tissues were also observed in FRO-derived spheres. The progressive increase in EpICD signal in opposite to a reduction of EpEx signal from the outer surface to the inner core of the spheres could indicate the existence of a radial gradient of EpCAM cleavage, probably due to the radial gradient of nutrients and oxygen typical of tumor spheres [9, 54] and the consequent progressive loss of epithelial differentiation towards a more mesenchymal phenotype [55–58] (Figs. 2 and 3).

Thanks to the manipulation of EpCAM cleavages in vitro through TAPI-2, BACE, DAPT and DIP treatments for the generation of 3D spheres, we observed that the integrity of EpCAM in the initial phases of the generation of the 3D spheres could be an advantage for putative tumor-initiating cells that are responsible for the organization of the 3D structure, especially in the very early stages (Fig. 3C, D). EpCAM role as an adhesion molecule [59–63], that could promote and intensify cell-cell contact, is guaranteed when the protein is in its full length form. The cleavage of the protein may occur in a subsequent phase as the sphere grows and cells adapt to variations in growth conditions and/or to the microenvironment within the spheres, as we observed at Immunofluorescence on FRO-derived spheres (Fig. 3B). In particular, the presence of pseudo-hypoxic condition induced by DIP treatment promotes the proteolytic activity of ADAM17 and γ-secretase [41, 48, 64–66], that are the main proteases involved in EpCAM cleavage, and the EpICD fragment may be released into the cytoplasm and/or nucleus.

With PLX-4032 treatment, our data demonstrated that the presence of EpCAM may be considered an additional factor that contribute to drug resistance in putative TICs. In a recently published paper, has been described that PLX-4032 may modulate thyroid CSCs survival. Immortalized cells that survived PLX-4032 treatment displayed increased frequency of aldehyde dehydrogenase (ALDH)-positive cells and thyrospheres formation abilities [67]. In agreement with this, our experiments confirmed that EpCAM + cells appeared to be more resistant than EpCAM- cells and this is a common feature of tumor cells that display tumor-initiating properties. We also noticed that PLX-4032 treatment seems to induce morphological changes of cells displaying a more elongated, “mesenchymal-like” shape in FRO cells, confirmed by the Aspect Ratio (A_R_) analysis of cell morphology (Fig. 4D). We postulate that this morphological change may be due to the activation of drug-escape mechanisms, consistently the induction of EMT is known to be one of the mechanisms of drug resistance in TICs [68–70].

While knowing that the spatial organization of cells in 3D spheres is a key factor in blocking drug penetration, we showed that the organization into 3D spheres is not sufficient per se to induce drug resistance, as FTC133 cells (completely EpCAM negative) are sensitive to PLX-4032 even if they generate spheres comparable in dimensions and morphology to FRO, which instead are resistant to the treatment (Fig. 4). Interestingly, FRO 3D spheres are mostly constituted by EpCAM + cells with different degrees of protein cleavage. Thus, we propose the variable patterns of EpCAM cleavage as additional fundamental events involved in TICs survival and drug resistance induction.

Further studies need to be performed to translate these results into patient-derived primary tumors to validate the findings on EpCAM observed in immortalized TC cell lines and expand the cohort of primary tumors. In addition, we will further characterize thyroid TICs, isolated through clonality assays and 3D sphere-forming assays, to evaluate the interaction between EpCAM and other known TICs markers for modulating the response to TKIs.

## Conclusions

Our study demonstrates that EpCAM expression and cleavage is playing a significant role in the biology of putative thyroid TICs and may play a fundamental role in the switch to a drug-resistant phenotype of UTCs cells. Until now, in vitro studies on the possible involvement of EpCAM in UTC pathology and tumor progression are generally few and do not investigate the role of its cleavages’ nor the tumorigenic potential of the EpICD fragment. Our 3D spheres model is representative of the variability of EpCAM expression observed in tissue samples, with the different gradient of EpCAM cleavages that corresponds to different areas of the tumor sections. We believe that the presence of EpCAM and its cleavage in a 3D organization could be an additional factor for tumor cells to induce drug resistance, alongside other mechanism of drug escape that may occur. In this sense, we propose EpCAM evaluation as a tool with a useful potential in the clinical decisions regarding patients’ therapy, particularly the EpICD domain could be considered a promising target for the development of new therapeutic options.

### Electronic supplementary material

Below is the link to the electronic supplementary material.

Supplementary Material 1

Supplementary Material 2

Supplementary Material 3

## Data Availability

The datasets analyzed during the current study are available in the Zenodo repositoryhttps://zenodo.org/doi/10.5281/zenodo.10953392.

## Abbreviations

ALDH: Aldehyde dehydrogenase
ATC: Anaplastic thyroid cancer
CSCs: Cancer stem-like cells
DIP: 2,2’-Bipyridyl
EGF: Epidermal growth factor
EGFR: Epidermal growth factor receptor
ELDA: Extreme limiting dilution analysis
EMT: Epithelial to mesenchymal transition
EpCAM: Epithelial cell adhesion molecule
EpEx: Extracellular domain of EpCAM
EpICD: Intracellular domain of EpCAM
FGF: Fibroblast growth factor
FTC: Follicular thyroid cancer
PDTC: Poorly differentiated thyroid cancer
Poly-hema: Poly(2-hydroxyethyl methacrylate)
PTC: Papillary thyroid cancer
RIP: Regulated intramembrane proteolysis
TC: Thyroid cancer
TICs: Tumor-initiating cells
TMD: Transmembrane domain
TKI: Tyrosine kinase inhibitor
UTCs: Undifferentiated thyroid cancers
