# Supplementary material for: Characterization of EpCAM in thyroid cancer biology by three-dimensional spheroids in vitro model
Source: Cancer Cell Int. 2024 Jun 4;24:196. doi: 10.1186/s12935-024-03378-2 (PMC11149206; doi:10.1186/s12935-024-03378-2)
Supplement: Supplementary file 1 — Supplementary Material 1 [file 12935_2024_3378_MOESM1_ESM.docx]

**Supplemental Methods**

***Light microscopy for 3D spheres dimensions and morphology***

Bright-field images of TC cell line-derived 3D spheres were obtained with a Leica Microsystems camera mounted on Leica MC 120 HD light microscope, at 5X magnification. The area of each sphere was calculated on Fiji Software by applying the ellipses formula. An average of the measurements of the area of at least 7 spheres for each independent replicate was reported.

***Fluorescence and Confocal microscopy***

Images were acquired with Nikon EclipseTi-E inverted microscope with Nikon INTENSILIGHT C-HGFIE fluorescent lamp and IMA10X Argon-ion laser System by Melles Griot. Fluorescent microscopy was applied for 2D adherent cells images acquisition with CFI Plan Apo VC 20X (Nikon) objective; at least 10 different fields were acquired for each condition for each cell line. Confocal microscopy was applied for tissue sections and 3D sphere images acquisition. Z-stacks were acquired with CFI Plan Apo VC 20X objective for healthy tissue and 40X Oil (Nikon) objective for PTC tissue. At least 10 different fields were acquired for both healthy tissue and PTC sections. 3D spheres images were acquired with CFI Plan Apo VC 40X Oil (Nikon) objective. Z-series acquisition was performed for 3D spheres images and 3D structure was reconstructed with Nikon NIS-Elements AR software. For each acquisition, 17 stack (2 μm z-step) were acquired, comprising the whole thickness of the sphere. For FRO-derived 3D spheres experiments a total number of 27 spheres were acquired with 40X magnification.

***Transient transfection and treatments with EpCAM cleavage’s inhibitors***

FRO and HEK 293T were transiently transfected with 1 μg of C-terminal EGFP [enhanced GFP (green fluorescent protein)]-tagged human EpCAM, a kind gift of professor B.G. Giepmans (University of Groningen, The Netherlands) ^1-3^, using FuGENE® HD transfection reagent (Promega Corporation) in Opti-MEM™ (Gibco). Treatments with EpCAM cleavages’ inhibitors were performed 48 hours after transfection. The inhibitors used are TAPI-2 (Merck Millipore), BACE (Merck Millipore), and DAPT (Merck Millipore). Pseudo-hypoxia was induced with 2,2’-Bipyridyl (DIP) (Merck Millipore). Proteins extraction was performed after 24 hours.

**References:**

1. Litvinov SV, Balzar M, Winter MJ, et al. Epithelial Cell Adhesion Molecule (Ep-CAM) Modulates Cell–Cell Interactions Mediated by Classic Cadherins. J Cell Biol 1997;139(5):1337–1348.

2. Schnell U, Kuipers J, Giepmans BNG. EpCAM proteolysis: new fragments with distinct functions? Biosci Rep 2013;33(2):e00030; doi: 10.1042/BSR20120128.

3. Winter MJ, Cirulli V, Briaire-de Bruijn IH, et al. Cadherins are regulated by Ep-CAM via phosphaditylinositol-3 kinase. Mol Cell Biochem 2007;302(1–2):19–26; doi: 10.1007/s11010-007-9420-y.
