# Supplementary material for: Characterization of EpCAM in thyroid cancer biology by three-dimensional spheroids in vitro model
Source: Cancer Cell Int. 2024 Jun 4;24:196. doi: 10.1186/s12935-024-03378-2 (PMC11149206; doi:10.1186/s12935-024-03378-2)
Supplement: Supplementary file 2 — Supplementary Material 2 [file 12935_2024_3378_MOESM2_ESM.docx]

**
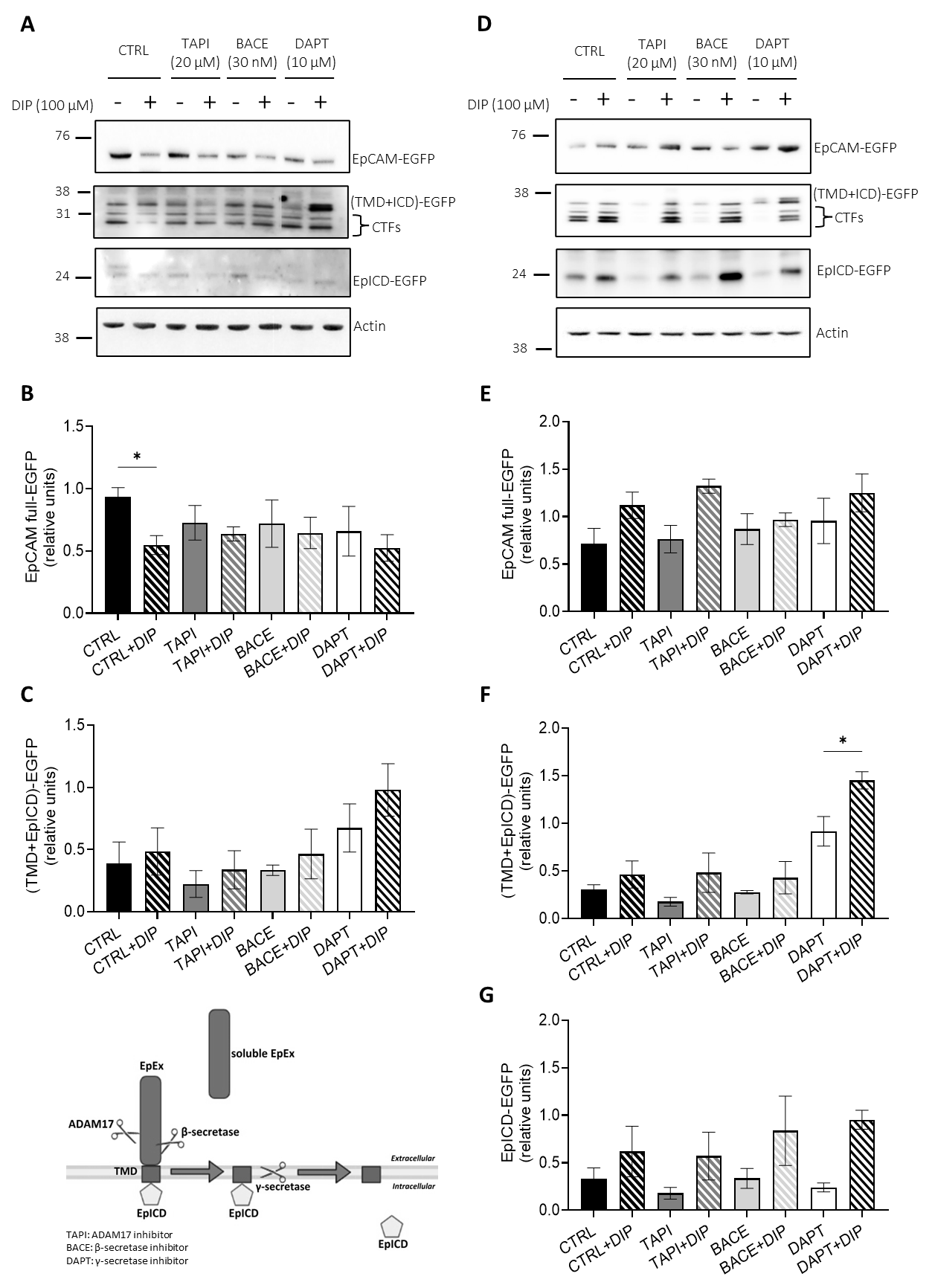
**

**Figure S1. Characterization of EpCAM cleavages in transfected FRO and HEK293T.**

Since very faint bands of lower molecular weight respect to EpCAM full length were detected in FRO adherent cells at Western Blot, we transiently transfected cells with a C-terminal EGFP-tagged EpCAM plasmid for a better characterization. Transfected cells were treated with selected concentrations of the different inhibitors of EpCAM cleavage TAPI-2 (20 μM), BACE (30 nM) and DAPT (10 μM). DIP (100 μM) was also added to mimic pseudo-hypoxic condition that may induce EpCAM shedding. (A) Thanks to the inhibition of the different proteases, we were able to identify different cleavage bands with an increase in molecular weight of around 20-25 kDa due to the EGFP tag: the full length protein of 67/68 kDa, the TMD plus EpICD fragment (TMD+EpICD) of 35/36 kDa and the EpICD fragment alone of 25/26 kDa. We also observed other cleavage bands with molecular weight between 30 and 34 kDa, called C-terminal fragments (CTFs), whose expression remained unchanged despite treatments with inhibitors, suggesting that probably other proteases may be involved. (B,C) As expected, full length EpCAM had the tendency to decrease after DIP treatment in all conditions respect to the treatments without DIP, while the TMD+EpICD fragment increased after DIP treatment in every condition, especially after inhibition of γ-secretase with DAPT. Since the EpICD signal was hard to detect clearly, we decided to transfect and treat with EpCAM cleavages’ inhibitors also HEK293T cells. Schnell and colleagues [44] first used this cell line to characterize EpCAM cleavages *in vitro* since HEK293T are easily transfectable. We decided to reproduce this *in vitro* model also to better identify the different bands obtained in FRO. (D) After treating transfected cells with different inhibitors in the same way as FRO, we were able to visualize the full length, the TMD+EpICD and the EpICD fragment alone. (E,F) Increased expression of both EpCAM full length and TMD+EpICD fragment after DIP treatment was observed in every condition. We obtained a significant increase of TMD+EpICD signal after inhibition of γ-secretase with DAPT and in pseudohypoxic condition induced by DIP. (G) As a further confirmation of the role of pseudohypoxia in improving the cleavage of EpCAM, we observed that the expression of the EpICD fragment alone is increased after DIP treatment in every condition. CTRL= untreated control transfected with human C-terminal EGFP. Statistical analysis: Non-parametric Mann-Whitney U test. *p <0.05.

**References**

44. Schnell U, Kuipers J, Giepmans BNG. EpCAM proteolysis: new fragments with distinct functions? Biosci Rep. 2013 Mar 19;33(2):e00030.

**
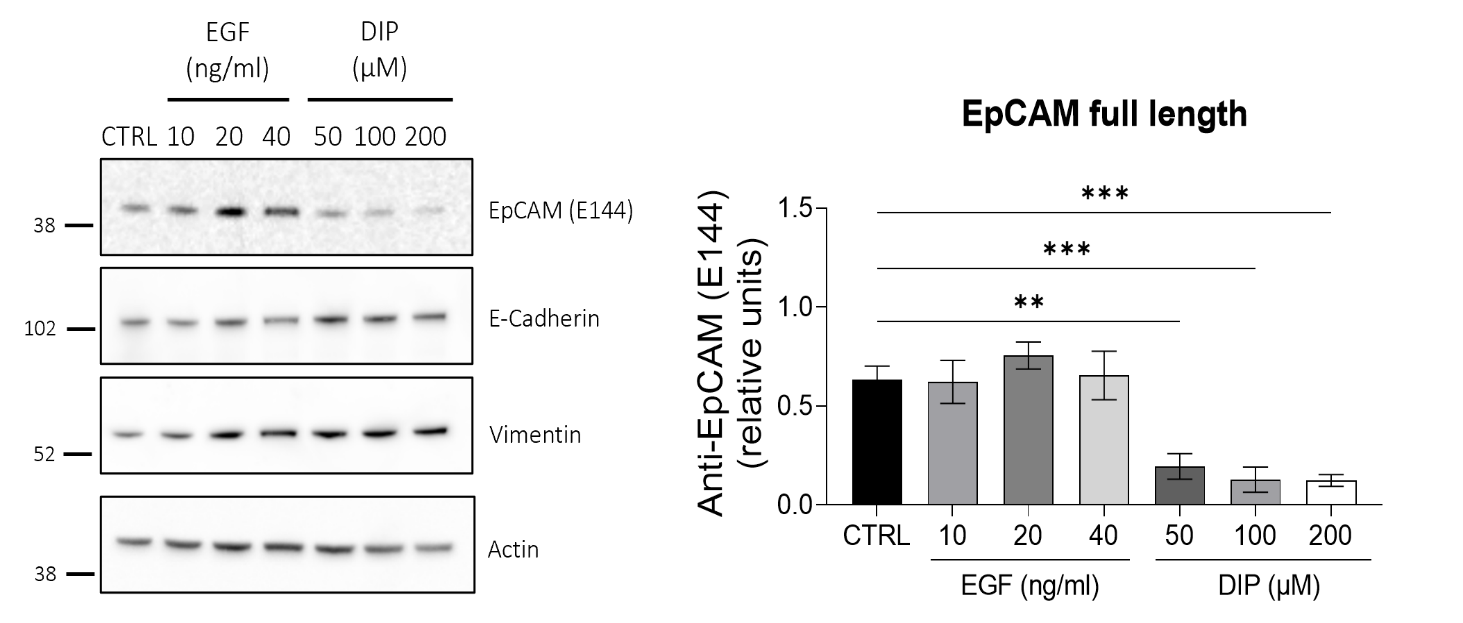
**

**Figure S2. Pseudo-hypoxia due to DIP treatment significantly affects EpCAM full length expression in FRO adherent cells.**

To study if and how the growing conditions of the spheres or the 3D organization alone may affect EpCAM expression and cleavage, FRO adherent cells in basal condition were treated with increasing concentrations of epidermal growth factor (EGF), since is one of the main growth factors used for 3D sphere maintenance, at selected concentrations (10 ng/ml, 20 ng/ml and 40 ng/ml) and increasing concentrations of 2,2’-Bipyridyl (DIP, 50 μM, 100 μM and 200 μM), used as an hypoxia-mimetic compound. Western blot of protein extracts was performed 24 h after treatments and relative expression of EpCAM, E-Cadherin and Vimentin was evaluated. We observed that EGF, at the concentration of 20 ng/ml which is the exact concentration applied for Sphere Medium, is associated with slight but not significant increase of EpCAM full length expression. On the other hand, increasing concentrations of DIP induced a significant reduction of EpCAM full length expression. We also observed that treatments with either EGF and DIP did not affect in a significant manner the expression of E-Cadherin and Vimentin. Actin was used as loading control. CTRL= untreated control. Statistical analysis: One way ANOVA followed by Dunnett’s multiple comparisons test. ***p <0.001, **p <0.01.
