# Supplementary material for: Characterization of EpCAM in thyroid cancer biology by three-dimensional spheroids in vitro model
Source: Cancer Cell Int. 2024 Jun 4;24:196. doi: 10.1186/s12935-024-03378-2 (PMC11149206; doi:10.1186/s12935-024-03378-2)
Supplement: Supplementary file 3 — Supplementary Material 3 [file 12935_2024_3378_MOESM3_ESM.docx]

**Table S1. Primary and secondary antibodies applied for Western Blot experiments.**

Primary (A) and secondary (B) antibodies used for Western Blot experiments with incubation time and respective dilutions.

(A)

| **Blocking Solution**  **(1 h R.T.)** | **Antibody**  **(o/n 4°C)** | **Concentration used** | **Productor** | **Expected molecular weight** |
| --- | --- | --- | --- | --- |
| 5% milk TBS-T | Anti-EpCAM Ab [E144] | 1:1000 | Abcam (ab32392) | ̴ 40 kDa |
| 5% milk TBS-T | Anti- EpCAM/TROP-1 Ab | 1:1000 | R&D (AF960) | ̴ 40 kDa |
| 5% milk TBS-T | Anti-GFP Ab | 1:2000  1:5000 | Abcam (ab290) | ̴ 20 kDa |
| 5% milk TBS-T | Anti-E-Cadherin Ab [M168] | 1:1000 | Abcam (ab76055) | 135 kDa |
| 5% milk TBS-T | Recombinant Anti-Vimentin Ab [EPR3776] | 1:2000  1:4000 | Abcam (ab92547) | 57 kDa |
| 5% milk TBS-T | Purified Mouse Anti-Actin Ab 5 | 1:1000 | BD Biosciences | 42 kDa |

(B)

| **Solution** | **Antibody**  **(1 h R.T.)** | **Concentration used** | **Productor** |
| --- | --- | --- | --- |
| 5% milk TBS-T | Goat Anti-Rabbit IgG Antibody (H+L) HRP | 1:5000 | SigmaAldrich (Merck Millipore) |
| 5% milk TBS-T | Goat Anti-Mouse IgG Antibody (H+L) HRP | 1:5000 | SigmaAldrich (Merck Millipore) |
| 5% milk TBS-T | Donkey Anti-Goat IgG Antibody (H+L) HRP | 1:5000 | Thermo Fisher |

**Table S2. Schematic description of the Immunofluorescence protocol applied and the primary and secondary antibodies used for the experiments.**

(A) Immunofluorescence protocol for adherent cells, 3D spheres and patient-derived tissues. Primary (B) and secondary (C) antibodies used for Immunofluorescence experiments with respective dilutions.

(A)

| **Immunofluorescence protocol** | **Adherent cells** | **3D spheres** | **Tissues** |
| --- | --- | --- | --- |
| Inclusion | **/** | **/** | O.C.T. solution (Bio Optica) |
| Cut | **/** | **/** | 10 μm thick cryosections on SuperFrost® slides |
| Wash PBS | x2 (5 min each) | x3 (5 min each) | x3 (5 min each) |
| Fixation | 4% PFA 10 min | 4% PFA 20 min | 4% PFA 30 min |
| Wash PBS | x2 (5 min each) | x3 (5 min each) | x3 (5 min each) |
| Permeabilization | 0.2% saponin in PBS 10 min | 0.2% saponin in PBS 15 min | 0.3% TritonTM X-100 10 min |
| Wash PBS | x2 (5 min each) | x3 (5 min each) | x3 (5 min each) |
| Blocking | 5% BSA in PBS 1h R.T. | 5% BSA in PBS 1h R.T. | Serum-free protein block (DAKO) 30 min R.T. |
| Primary Antibody incubation | o/n 4°C in the dark | o/n 4°C in the dark | o/n 4°C in the dark |
| Wash PBS | x2 (5 min each) | x3 (5 min each) | x3 (5 min each) |
| Secondary Antibody incubation | 5% BSA in PBS solution 1h R.T. | 5% BSA in PBS solution 1h R.T. | 5% BSA in PBS solution 1h R.T. |
| Wash PBS | x2 (5 min each) | x3 (5 min each) | x3 (5 min each) |
| DAPI solution | / | 2 µg/ml in H2O 20 min | / |
| Wash H2O | / | x2 | / |
| Mounting | 15 μl Vectashield Hard-Set mounting medium with DAPI | 15 μl Vectashield Hard-Set mounting medium with DAPI | 25 μl Vectashield Hard-Set mounting medium with DAPI |

(B)

| **Blocking Solution** | **Antibody** | **Concentration used** | **Productor** |
| --- | --- | --- | --- |
| 5% BSA PBS | Anti-EpCAM Ab [E144] | 1:150 | Abcam (ab32392) |
| 5% BSA PBS | Anti- EpCAM/TROP-1 Ab | 1:150 | R&D (AF960) |
| 5% BSA PBS | Wheat Germ Agglutinin (WGA) | 5 μg/ml | Thermo Fisher Scientific |

(C)

| **Solution** | **Antibody** | **Concentration used** | **Conjugated fluorophore** | **Productor** |
| --- | --- | --- | --- | --- |
| 5% BSA PBS | Donkey anti-Rabbit IgG (H+L) Secondary Ab | 1:500 | Alexa Fluor™ 555 | Thermo Fisher Scientific |
| 5% BSA PBS | Donkey anti-Goat IgG (H+L) Secondary Ab | 1:500 | Alexa Fluor™ 488 | Thermo Fisher Scientific |

**Table S3. Proliferation curves parameters reported as best-fit values.**

|  |  | **Vemurafenib (PLX-4032)** | |
| --- | --- | --- | --- |
|  |  | **2D adherent cells** | **3D spheres** |
| FRO (ATC) | Bottom | 5.70 | 90.87 |
|  | IC50 | 4.97 | 2.37 |
|  | Slope | -0.64 | -1.42 |
| HTCC3 (PDTC) | Bottom | 12.85 | 41.35 |
|  | IC50 | 9.26 | 16.89 |
|  | Slope | -0.84 | -0.56 |
| FTC133 (ATC) | Bottom | 10.19 | 25.03 |
|  | IC50 | 36.49 | 42.68 |
|  | Slope | -1.82 | -3.80 |
